# Pedogenetic processes in anthrosols with pretic horizon (Amazonian Dark Earth) in Central Amazon, Brazil

**DOI:** 10.1371/journal.pone.0178038

**Published:** 2017-05-23

**Authors:** Rodrigo S. Macedo, Wenceslau G. Teixeira, Marcelo M. Corrêa, Gilvan C. Martins, Pablo Vidal-Torrado

**Affiliations:** 1 Soil Science Department, University of São Paulo, Piracicaba, São Paulo, Brazil; 2 Embrapa Solos, Rio de Janeiro, Rio de Janeiro, Brazil; 3 Universidade Federal Rural de Pernambuco, Unidade Acadêmica de Garanhuns, Garanhuns, Pernambuco, Brazil; 4 Embrapa Amazônia Ocidental, Manaus, Amazonas, Brazil; RMIT University, AUSTRALIA

## Abstract

Anthrosols known as Amazonian Dark Earth (ADE) have borne witness to the intensification of sedentary patterns and the demographic increase in Central Amazon. As a result, a recurring pattern has been observed of mounds with ADE arising from domestic activities and the disposal of waste. The objective of this research was to demonstrate the relationship of these anthropic activities with pedogenetic formation processes of ADE in the municipality of Iranduba, Brazil. Disturbed and undisturbed soil samples were taken from two areas of ADE (pretic horizon) and from a non-anthropic pedon. Physical, chemical, micromorphological and SEM-EDS analyses were performed. The coarse material of the pretic horizons consisted predominantly of quartz, iron nodules, ceramics and charcoal fragments, and the fine material is organo-mineral. There was a direct relationship between the color of pretic horizons and the number of charcoal fragments. The thickness of the ADE results from the redistribution of charcoal at depth through bioturbation, transforming subsurface horizons into anthropic horizons. ADE presents granular microaggregates of geochemical and zoogenetic origin. Degradation of iron nodules is intensified in pretic horizons, promoting a reverse pedogenic process contributing to the xanthization process. Surprisingly the anthropic activities also favor clay dispersion and argilluviation; clay coatings on the ceramic fragments and in the pores demonstrate that this is a current process. Processes identified as contributing to ADE genesis included: i) addition of organic residues and ceramic artifacts (cumulization) with the use of fire; ii) mechanical action of humans, roots and macrofauna (bioturbation); iii) melanization of deeper horizons as a result of bioturbation; iv) argilluviation and degradation of iron nodules. This study offers new support to archaeological research in respect to ADE formation processes in Central Amazon and confirmed the hypothesis that ancient anthropic activities may trigger and/or accelerate pedogenetic processes previously credited only to natural causes.

## Introduction

The low cation exchange capacity of the upland soils in Central Brazilian Amazon is attributed to the predominance of weathered soils with low levels of organic matter (OM). The low OM content is a result of the combination of high temperatures and a moisture regime favoring its decomposition [1]. In the midst of these weathered soils of low fertility, there are horizons enriched in Ca^2+^, Mg^2+^, P, Zn^+^ and Mn^+^, known regionally as Amazonian Dark Earth (ADE) [2], [3], [4]. They occur predominantly as surface horizons associated with Ferralsols, Acrisols, Lixisols, Plinthosols, Fluvisols and Cambisols [5], corresponding to the recently created pretic horizon among Anthrosols on the World Reference Base of Soil Resources [6].

Due to its favorably contrasting chemical characteristics in relation to surrounding soils, ADE has been the subject of multidisciplinary studies seeking to understand its formation, even when subjected to intense agricultural practices. Micromorphological studies on ADE in Central Amazon have shown that its high P and Ca^2+^ contents arise from microscopic bone fragments and that anthropic horizons show organo-mineral aggregates with a mixture of A horizon materials and mineral aggregates of subsurface horizons [3]. Micromorphology associated with scanning electron microscopy (SEM) coupled with electron-dispersive X-rays (EDS) in ADE were able to identify the forms of the elements in ADE [7], the chemical composition of the soil matrix and the added material [3], [8], as well as pedogenetic changes due to bone fragments and the neoformation of minerals [8].

Currently, anthropic addition of organic and inorganic substances allied to the activity of soil fauna is the most accepted model for the formation of ADE [9], [10]. In relation to pedogenetic processes, previous studies have shown that darkening of the soil (melanization), resulting from high levels of organic material, pyrogenic carbon and manganese oxides and anthropedoturbation—homogenization of the soil through biological and human action—are important processes for ADE formation occurring in Acrisols at the Hatahara site, Amazônia Central—Brazil [3], [10], [11], [12], [13], [14]. In turn, Acrisols in the region present evidence of argilluviation under natural conditions [10], demonstrating the clay migration process from the A horizon (eluvial) to the B horizon (illuvial), forming a subsurface textural B horizon (Bt). Lastly, it is common to find ferruginous concretions arising from Lateritic mantle in Acrisols and Ferrasols of the region [15], which may be found in degradation due to the current environmental conditions [16].

A detailed archaeological characterization is essential in studies on ADE, contributing to the understanding of the use of resources and changes in pre-Columbian periods in Amazonian communities [17], [18]. The archaeological information currently available shows that the ADE formation period in the central region of the Amazon was a moment of the intensification of sedentary settlement patterns and demographic increase, possibly combined with greater use of subsistence farming strategies [19].

Therefore, recurring pattern of dark earth has been identified in a large area of the Amazonian region, including the Caldeirão archeological site in Central Amazon, the location of this study. This pattern consists of mounds in the form of a ring surrounding circular or semi-circular terraces of approximately 10–20 m in diameter [20]. These authors showed that domestic activities related to houses or yards were concentrated on these terraces, while the mounds were formed from refuse middens. It has recently been shown that the disposal and/or management of plants of recognized use by pre-Colombian people, notably species of Arecaceae, was also an important activity carried out on these mounds [21].

Despite the afore mentioned and scientific interest in ADE, there is still a lot of debate and very little concrete knowledge in respect to specific processes and contexts of its formation [20]. Specifically in the Caldeirão site, questions such as whether or not the mounds were formed through the overlap of waste disposal zones with cultivation and if there is a pattern in the disposal of waste, are yet to have been answered. Based on current knowledge in respect to ADE mounds on the Caldeirão site, this study is justified by the fact that it offers new pedological details to archeological postulations showing how anthropic activities influenced ADE formation. This site was chosen for its history in interdisciplinary research on these soils [20], [22], [23], [24] and because it presents typical anthropic soil horizons that provide information on key components and processes involved in soil genesis and evolution.

Therefore, the objective of this study was to identify the principal pedogenetic processes involved in ADE formation at Caldeirão, testing the hypothesis that ancient anthropic activities were installed and intensified pedogenetic processes naturally occurring in the soils. These results may support the updating of criteria for pretic horizon on the World Reference Base for Soil Resources [6] as well to contribute to the current debate on ADE formation and archaeological studies on the intensity of human impact on the Amazonian landscape.

## Materials and methods

### Study site

**Fig 1:**
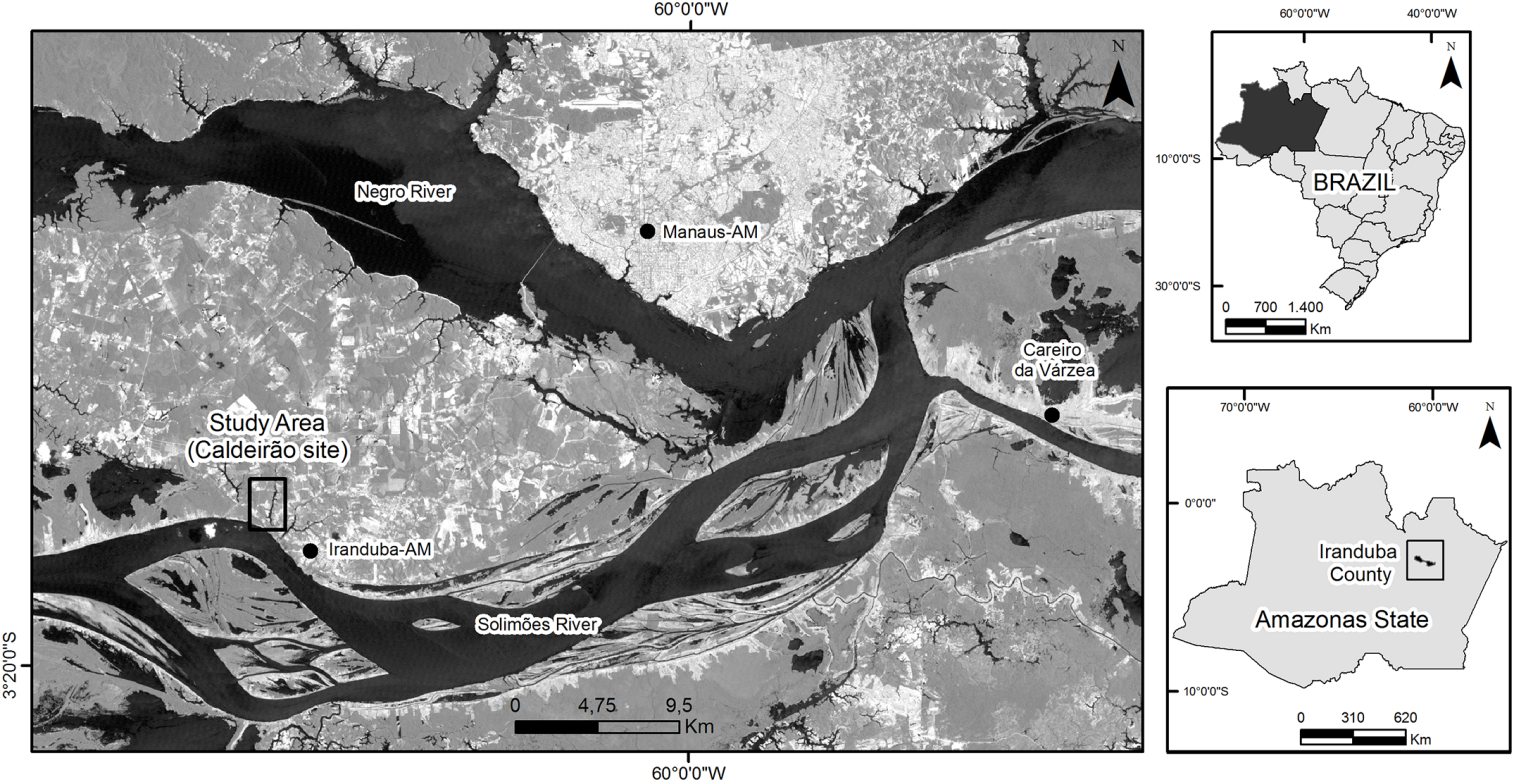
Experimental Station of Caldeirão, Central Amazon—Brazil. The base image is LANDSAT 5 image (2011). The Landsat images were downloaded from USGS.

The study was carried out under authorization of the head of the Embrapa Western Amazon Dr Luiz Marcelo Brum Rossi in the Experimental Research Station of Caldeirão (centered at 03°14’22”- 03°15’47” S and 60°13’02”- 60° 13’50” W) (Fig 1) in the municipality of Iranduba—Amazonas State—Brazil. The climate is tropical rainy, Aw type (Köppen classification). The average annual temperature is 26.7°C and average annual relative humidity ranges from 77 to 84%. The average annual precipitation is 2100 mm and is distributed in a rainy season, from November to May and another less rainy season, stretching over the other months of the year [25]. The site belongs to the Domain of morphostructural sedimentary basins and unconsolidated covers inserted into the dissected plateaus of the Negro–Uatumã Rivers and presents dissected relief with convex and tabular tops [26].

The soils at the site originated from weathering of sedimentary rocks from the Tertiary period of the Alter do Chão formation characterized by clayey and quartz sandstones, arkoses and argillites [25]. The predominant soils in the region are Xanthic Ferralsols in different textural classes, Plinthic Ferralsols, Pisoplinthic Plinthosols and Xanthic Acrisols [6], [25], [27].

### Soil description and sampling

**Fig 2:**
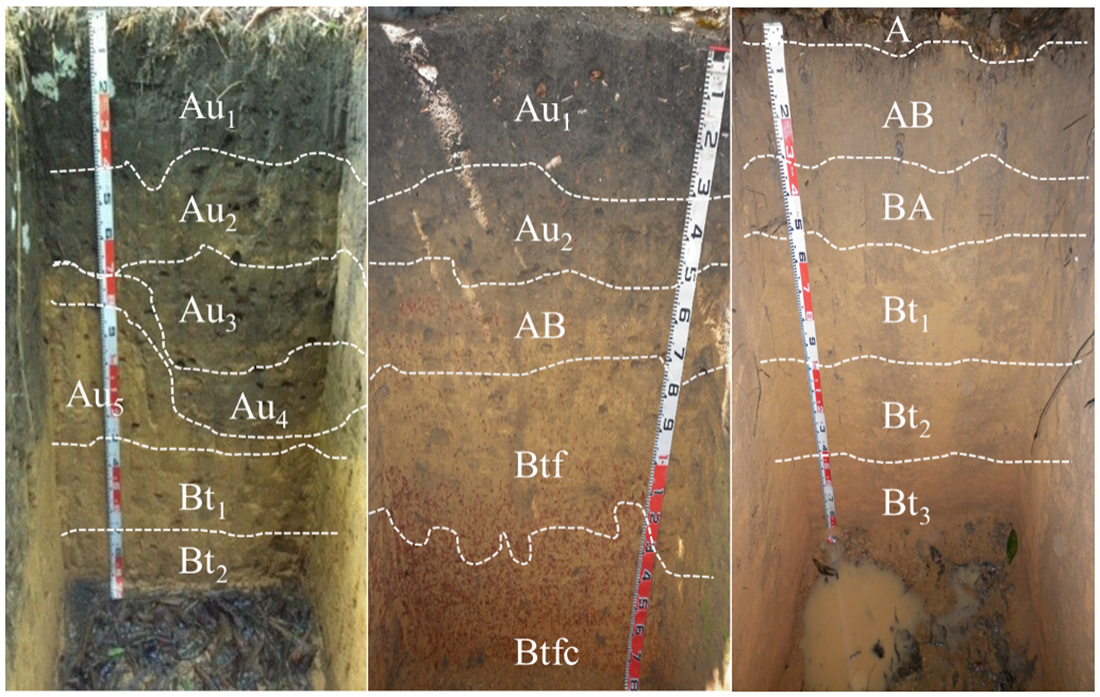
(A) Pretic Anthrosol (Orthodystric, Clayic) (P1); (B) Pretic Anthrosol (Lixic, Orthoeutric, Clayic) (P2); (C) Haplic Xanthic Acrisol (Hyperdystric, Clayic) (P3). Central Amazon—Brazil.

In order to obtain soil profiles, two sites were selected reflecting the difference between the ADE and the surrounding soil (non-anthropic). The areas can be found beneath secondary forest, in similar topographic positions, in an area not subjected to flooding (solid ground) at the margins of the Amazonas River. Two profiles with anthropic horizons—ADE (P1 and P2) and a non-anthropic profile (P3) (Fig 2) were described according to their morphological characteristics, sampled [28] and classified according to WRB [6]. Disturbed samples were collected, for each horizon identified in the field, for physical and chemical soil analyses. Undisturbed samples were collected from each of the following horizons: Au_1_, Au_2_, Au_3_, Au_4_, Au_5_ and Bt_1_ (P1), Au_1_, Au_2_, AB e Btcf (P2) e AB, BA/Bt_1_, Bt_1_ e Bt_2_ (P3) for micromorphology, scanning electronic microscopy (SEM) and electron-dispersive x-rays (EDS). These samples were collected using paper card boxes with dimensions of 12x7x4 cm.

### Physico-chemical analyses

Sand fractions were obtained through wet sieving and silt (0.002–0.053 mm) and clay (<0.002 mm) contents were estimated by sedimentation. The pH were determined in water and KCl 1 mol L^-1^ (1:2.5 v/v); organic carbon was determined after oxidation with potassium dichromate (K_2_Cr_2_O_7_) 0.4 mol L^-1^; Ca^2+^, Mg^2+^ and Al^3+^ were extracted with KCl 1 mol L^-1^; potential acidity (H + Al) were extracted with calcium acetate 0.5 mol L-1—pH 7.0; and P, K^+^ and Na^+^ were extracted in a solution of H_2_SO_4_ 0.0125 mol L^-1^ + HCl 0.05 mol L^-1^ (Mehlich-1). Concentrations of Ca^2+^ and Mg^2+^ were determined by atomic absorption spectroscopy, K^+^ and Na^+^ by flame photometry, Al^3+^ and H + Al by complexometric titration and P by colorimetry. Methodological details are described by Embrapa [29].

### Micromorphology

Undisturbed soil samples were dried in an oven at 40°C to remove residual moisture. Impregnation was performed with polyester resin [30] by adding styrene monomer. In the sand fraction, magnetic minerals were separated by a hand magnet. Magnetite was compared with that observed in the examination of the thin sections. The description of the textural pedofeatures, grain size estimation and other micromorphological attributes were performed in accordance with Bullock et al. [31].

### Scanning electronic microscopy (SEM) and electron-dispersive X-rays (EDS)

These analyses were carried out in order to semi-quantify mineral components and identify the main forms of the selected components. The microanalyses were obtained by submitting the thin sections to SEM-FEI Inspect S 50 coupled to EDS.

## Results

### Soil morphology

Profiles P1 and P2 contained pretic horizons with a pronounced presence of charcoal fragments. The coarse fraction (> 2 mm) of the pretic horizons contained petroplinthic nodules, charcoal and ceramic fragments. The coarse fraction of Btf and Btfc horizons consisted of reddened plinthites (2.5YR 4/8 e 10YR 5/8) and ferruginous nodules (petroplinthites), respectively.

The diameter of ceramic fragments increased with depth from Au_1_ to Au_3_ (P1). At the base of Au_3_, the ceramic fragments were aligned with the charcoal fragments. Alignment of charcoals without ceramics occurred at the concave base of Au_4_. The pretic horizon of P2 presented a lower content of charcoal fragments and a lower amount of ceramic fragments compared to P1. In general, ceramic fragments showed aspects of charring, implied by very dark brown mottles (10YR 2/2).

The ADE has a thickness of 130 cm in P1 and 48 cm in P2. The non-anthropic A horizon (P3) has a thickness of 5 cm. The color of pretic horizons from P1 ranged from black (10YR 2/1—Au_1_) to dark yellowish brown (10YR 4/4 –Au_4_ and Au_5_), while the Au_1_ and Au_2_ (P2) ranged from black (10YR 2/1) to very dark gray (10YR 3/1) (Table 1). These colors are common in ADE [3], [4], [6]. The non-anthropic A horizon is dark yellowish brown (10YR 3/4). All Bt horizons range from yellowish brown (10YR 5/6) to strong brown (7.5YR 5/8). Mottles occur as dark yellowish brown in Au_2_ (P1) and AB (P2), and red (2.5YR 4/8) in the Bt horizons (P2).

**Table 1:** Some morphological and physical properties in (A) Pretic Anthrosol (Orthodystric, Clayic), (B) Pretic Anthrosol (Lixic, Orthoeutric, Clayic) and (C) Haplic Xanthic Acrisol (Hyperdystric, Clayic). Central Amazon—Brazil.

Horizon	Depth (cm)	Color (moist)	Structure^a^	Gravel^b^			Sand	Silt	Clay	WDC^c^
				Ceramics	Carbon	Nodules	%			
***Pedon 1- Pretic Anthrosol (Orthodystric*, *Clayic)***										
**Au**_**1**_	0–40	10YR 2/1	w m sbl/w l gr	c l	f vl	f	57	10	33	7
**Au**_**2**_	40–71	10YR 3/1	w m sbl/w l gr	c l	f vl	r	58	5	37	5
**Au**_**3**_	71–90	10YR 3/3	w m sbl/ w l gr	f l	c l	f	52	2	46	5
**Au**_**4**_	90–105	10YR 4/4	md lg abl/md l gr	f lg	f m	f	43	3	54	5
**Au**_**5**_	105–130	10YR 3/4	st lg sbl/st l gr	-	a m	-	42	3	55	5
**Bt**_**1**_	130–165	10YR 5/6	md lg ab	-	-	-	27	3	70	2
**Bt**_**2**_	165–200	7,5YR 5/8	md lg abl	-	-	-	27	3	70	2
***Pedon 2—Pretic Anthrosol (Lixic*, *Orthoeutric*, *Clayic)***										
**Au**_**1**_	0–22	10YR 2/1	w l abl/w l gr	f l	c	f	54	3	43	12
**Au**_**2**_	22–48	10YR 3/3	md m abl	f lg	c	f	44	6	50	5
**AB**	48–60	10YR 4/6	md lg abl	-	-	c	40	5	55	5
**Btf**	60–100	10YR 5/4	md lg abl	-	-	a	27	3	70	2
***Pedon 3—Haplic Xanthic Acrisol (Hyperdystric*, *Clayic)***										
**A**	0–5	10YR3/4	w m sbl/w l gr	-	r	r	63	2	35	5
**AB**	5–38	10YR 4/3	w m sbl/w m gr	-	f	-	51	1	48	2
**BA**	38–60	10YR 4/4	w m sbl/w m gr	-	-	-	48	2	50	2
**Bt**_**1**_	60–110	10YR 5/6	md lg abl	-	-	-	35	5	60	2
**Bt**_**2**_	110–155	7,5YR 5/6	st lg abl	-	-	-	30	2	68	2
**Bt**_**3**_	155–200	5YR 5/6	st lg abl	-	-	-	29	1	70	2

^a^ Development: w = weak, md = moderate, st = strong. Size: l = little, m = medium, lg = large. Type: gr = granular, sbl = subangular blocky, abl = angular blocky

^b^ Particles > 2 mm: Amount: r = rare, f = few, c = common, a = abundant. Size: vl = very little, l = little, m = medium, lg = large

^c^ Water dispersible clay

In the Bt horizons of ADE, particularly in P1, there were abundant elongated/oval biological channels of medium and large size (Ø 1.5–3 cm) filled with dark friable microaggregates. Similarly, in the pretic horizons biological channels filled with friable aggregates from the Bt horizon were present. Pretic horizons and non-anthropic A horizon have weakly developed blocky structures that fall apart at weak points of small and very small granules. All subsurface horizons show large blocks with a moderate to strong degree of aggregation. Common moderate to strong and common weak clay skins occur in Bt_1_ horizons and Au_3_/Au_4_ (P1), respectively, while few weak clay skins occur in the subsurface horizons of P3.

### Physico-chemical attributes and soil classification

The textural class of pretic horizons in P1 ranged from sandy clay loam to clayey, while the pretic horizons in P2 are clayey. Comparing the AB horizons (P3) and Au_1_ (P1), the texture changed from clayey to sandy clay loam. The surface horizon of the non-anthropic soil has a texture of sandy clay loam and all Bt horizons are very clayey (Table 1).

Pretic horizons have strong to moderate acidity (pH 5.11–5.73), whereas non-anthropic A horizon is extremely acidic (pH 4.12) (Table 2). Ca^2+^ content is high in pretic horizons, mainly in P2, and the contents of Mg^2+^ and K^+^ are low. While Al^3+^ content in P3 is high, in pretic horizons these values are ≤ 0.40 cmol_c_ kg^-1^ (Table 2). The anthropic horizons, notably P1, exhibit greater P content. The Bt horizons of ADE profiles are enriched with Ca^2+^, Mg^2+^, K^+^ and P in relation to Bt horizons of P3.

**Table 2:** Chemical properties in (A) Pretic Anthrosol (Orthodystric, Clayic), (B) Pretic Anthrosol (Lixic, Orthoeutric, Clayic) and (C) Haplic Xanthic Acrisol (Hyperdystric, Clayic). Central Amazon—Brazil.

Hz	pH		K^+^	Na^+^	Ca^2+^	Mg^2+^	Al^3+^	H+Al	CEC^a^	BS^b^	m^c^	OC^d^	P
	H_2_O	KCl	cmol_c_ kg^-1^							%		%	mg kg^-1^
***Pedon 1- Pretic Anthrosol (Orthodystric*, *Clayic)***													
**Au**_**1**_	5.11	4.62	0.06	0.06	6.44	0.58	0.13	6.28	13.42	53.21	1.79	0.24	56
**Au**_**2**_	5.23	4.42	0.03	0.05	3.41	0.39	0.20	5.00	8.87	43.65	4.91	2.11	138
**Au**_**3**_	5.18	4.19	0.03	0.07	2.27	0.32	0.40	4.97	7.66	35.10	12.95	1.90	124
**Au**_**4**_	5.17	4.73	0.03	0.06	2.19	0.32	0.33	7.03	9.63	27.00	11.26	1.78	112
**Au**_**5**_	5.15	4.37	0.02	0.05	2.11	0.28	0.27	5.66	8.12	30.32	9.88	0.44	111
**Bt**_**1**_	5.01	4.38	0.03	0.08	1.96	0.29	0.20	4.69	7.04	33.42	7.83	0.36	83
**Bt**_**2**_	4.82	4.46	0.03	0.05	1.95	0.29	0.17	5.38	7.70	30.09	6.84	0.20	84
***Pedon 2—Pretic Anthrosol (Lixic*, *Orthoeutric*, *Clayic)***													
**Au**_**1**_	5.73	5.01	0.09	0.02	6.16	1.06	0.00	2.33	9.65	75.87	0.00	2.43	109
**Au**_**2**_	5.42	4.46	0.05	0.01	4.25	0.66	0.08	2.24	7.21	68.93	1.58	1.03	208
**AB**	5.43	4.44	0.04	0.02	3.84	0.39	0.03	2.01	6.30	68.10	0.69	0.37	268
**Btf**	4.91	4.43	0.05	0.02	2.64	0.33	0.13	2.48	5.52	55.05	4.10	0.17	232
***Pedon 3—Haplic Xanthic Acrisol (Hyperdystric*, *Clayic)***													
**A**	4.12	3.98	0.07	0.06	0.04	0.11	2.81	6.82	7.10	3.95	90.92	2.75	1
**AB**	4.24	4.17	0.03	0.01	0.03	0.04	1.52	5.95	6.06	1.83	93.18	1.42	1
**BA**	4.43	4.21	0.01	0.01	0.01	0.03	1.40	5.44	5.50	1.12	95.79	1.30	1
**Bt**_**1**_	4.95	4.39	0.01	0.01	0.02	0.03	1.35	4.77	4.84	1.41	95.19	0.93	1
**Bt**_**2**_	5.22	4.48	0.01	0.01	0.01	0.02	0.94	4.32	4.37	1.18	94.81	0.36	1
**Bt**_**3**_	5.24	4.64	0.01	0.00	0.01	0.02	0.92	4.33	4.37	1.02	95.38	0.24	1

^a^ Cation exchange capacity.

^b^ base saturation.

^c^ Al saturation.

^d^ organic carbon.

P1 and P2 were classified as Anthrosols because they show a pretic horizon with thickness > 50 cm. P3 was classified as Acrisol for presenting an argic horizon. Along with the characteristics mentioned above, the soils correspond to Pretic Anthrosol (Orthodystric, Clayic) (P1), Pretic Anthrosol (Lixic, Orthoeutric, Clayic) (P2) and Haplic Xanthic Acrisol (Hyperdystric, Clayic) (P3).

### Micromorphology and SEM-EDS

The coarse material of pretic horizons (Tables 3 and 4) and non-anthropic A horizon ranged from 35 to 45% (Table 5). The other horizons are comprised predominantly of fine quartz sand (100 μm) and magnetite traces. Charcoal occurs in all horizons with predominance and smaller size in pretic horizons. Ceramic fragments occur only in pretic horizons. Neither bone fragments nor burnt phytoliths were found in the anthropic horizons [12], [13].

**Table 3:** Summary of the micromorphological description of the Pretic Anthrosol (Orthodystric, Clayic). Central Amazon—Brazil.

Features	Au_1_	Au_2_	Au_3_	Au_4_	Au_5_	Bt_1_
**Groundmass**	1. Related distribution: single spaced porphyric and enaulic	Idem	Idem	Single and double spaced porphyric with minor enaulic	Idem	Open porphyric.
	2. B-fabric: undifferentiated	Cross striated b-fabric	Idem	Undifferentiated	Cross striated and mosaic-speckled	Grano/poroestriated, cross striated and mosaic-speckled
**Microstructure**	1. Peds: microaggregate and subangular blocky	Idem	Subangular blocky and microaggregate	Idem	Idem	Massive and subangular blocky
	2. Voids: channels, vughs, chambers and cracks	Idem	Channels, vughs, and cracks	Chambers, channels, vughs and cracks	Vughs, channels, chambers and cracks	Vughs, chambers and cracks
**Basic mineral components**	1. Coarse fraction: quartz (80%), ceramics (10%), charcoal (10%) and rare magnetite	Quartz (91%), charcoal (8%) and magnetite (1%)	Quartz (89%), charcoal (5%), ceramics (5%) and magnetite (1%)	Quartz (90%), charcoal (4%), ceramics (5%) and magnetite (1%)	Quartz (92%), charcoal (2%), ceramics (5%) and magnetite (1%)	Quartz (99%), charcoal (1%) e rare magnetites
	2. Fine fraction: isotropic organo-mineral. Dark gray (10YR 4/1)	Isotropic organo-mineral. 60% brownish yellow (10YR 6/8), 30% yellow (10YR 8/8) and 10% dark gray	Isotropic organo-mineral. 80% brownish yellow and 20% yellow	Isotropic organo-mineral. 45% brownish yellow, 35% dark gray and 20% yellow	Isotropic organo-mineral. 50% yellow, 35% dark gray and 10% brownish yellow	Anisotropic mineral. Yellow
**Pedofeatures**	1. Textural: microlaminated illuvial clay coatings	Laminated and non-laminated illuvial clay coatings	Microlaminated and non-laminated illuvial clay coatings	Idem	Non-laminated illuvial clay coatings	Microlaminated and non-laminated illuvial clay coatings; non-laminated hypo-coatings
	2. Amorphous: charcoal and iron nodule	Charcoal and iron nodule	Charcoal and iron nodule	Idem	Charcoal	None

**Table 4:** Summary of the micromorphological description of the Pretic Anthrosol (Lixic, Orthoeutric, Clayic). Central Amazon—Brazil.

Features	Au_1_	Au_2_	AB	Btfc
**Groundmass**	1. Related distribution: enaulic	Single spaced porphyric and enaulic	Enaulic	Open porphyric
	2. B-fabric: stipple-speckled	Stipple-speckled b-fabric	Reticulate striated and stipple-speckled	Grano/poro/parallel striated, mosaic and stipple-speckled
**Microstructure**	1. Peds: microaggregate and subangular blocky	Idem	Subangular blocky and microaggregate	Subangular blocky
	2. Voids: channels, chambers, cracks and vughs	Channels, vughs and cracks	Compound packing, channels, chambers and vughs	Channels, chambers, vughs and cracks
**Basic mineral components**	1. Coarse fraction: quartz (87%), charcoal (10%), ceramics (3%) and rare magnetite	Quartz (90%), charcoal (7%), ceramics (3%) and rare magnetite	Quartz (95%) and charcoal (5%)	Quartz (100%) and rare magnetite
	2. Fine fraction: isotropic organo-mineral. Dark gray	Isotropic organo-mineral. Yellowish brown (10YR 5/4)	Isotropic organo-mineral. Brown (7,5 YR 4/4)	Anisotropic mineral. Reddish yellow (7,5YR 6/8)
**Pedofeatures**	1. Textural: laminated illuvial clay coatings	Non-laminated illuvial clay coatings	Microlaminated and non-laminated illuvial clay coatings	Idem
	2. Amorphous: charcoal and iron nodule	Idem	Iron nodule	Idem

**Table 5:** This is the Table 5 Summary of the micromorphological description of the Haplic Xanthic Acrisol (Hyperdystric, Clayic). Central Amazon—Brazil.

Features	AB	BA/Bt_1_	Bt_1_	Bt_2_
**Groundmass**	1. Related distribution: double spaced porphyric	Idem	Idem	Idem
	2. B-fabric: mosaic and stipple-speckled b-fabric	Undifferentiated and stipple-speckled	Grano/cross/parallel striated, mosaic and stipple-speckled	Idem
**Microstructure**	1. Peds: subangular blocky and microaggregate	Subangular blocky	Idem	Idem
	2. Voids: channels, cracks and vughs	Channels, vughs, chambers and cracks	Vughs, chambers and cracks	Idem
**Basic mineral components**	1. Coarse fraction: quartz (98%), charcoal (2%) and rare iron nodules and magnetite	Quartz (98%) and charcoal (2%)	Quartz (98%), charcoal (2%) and rare magnetite	Idem
	2. Fine fraction: isotropic mineral. 60% brownish yellow and 40% yellow	Isotropic mineral. Yellowish red (5YR 5/6)	Isotropic mineral. Yellow (7,5 YR 4/4)	Isotropic mineral. Reddish yellow
**Pedofeatures**	1. Textural: none	Idem	Idem	Idem
	2. Amorphous: charcoal and iron nodule	Charcoal	None	Idem

**Fig 3:**
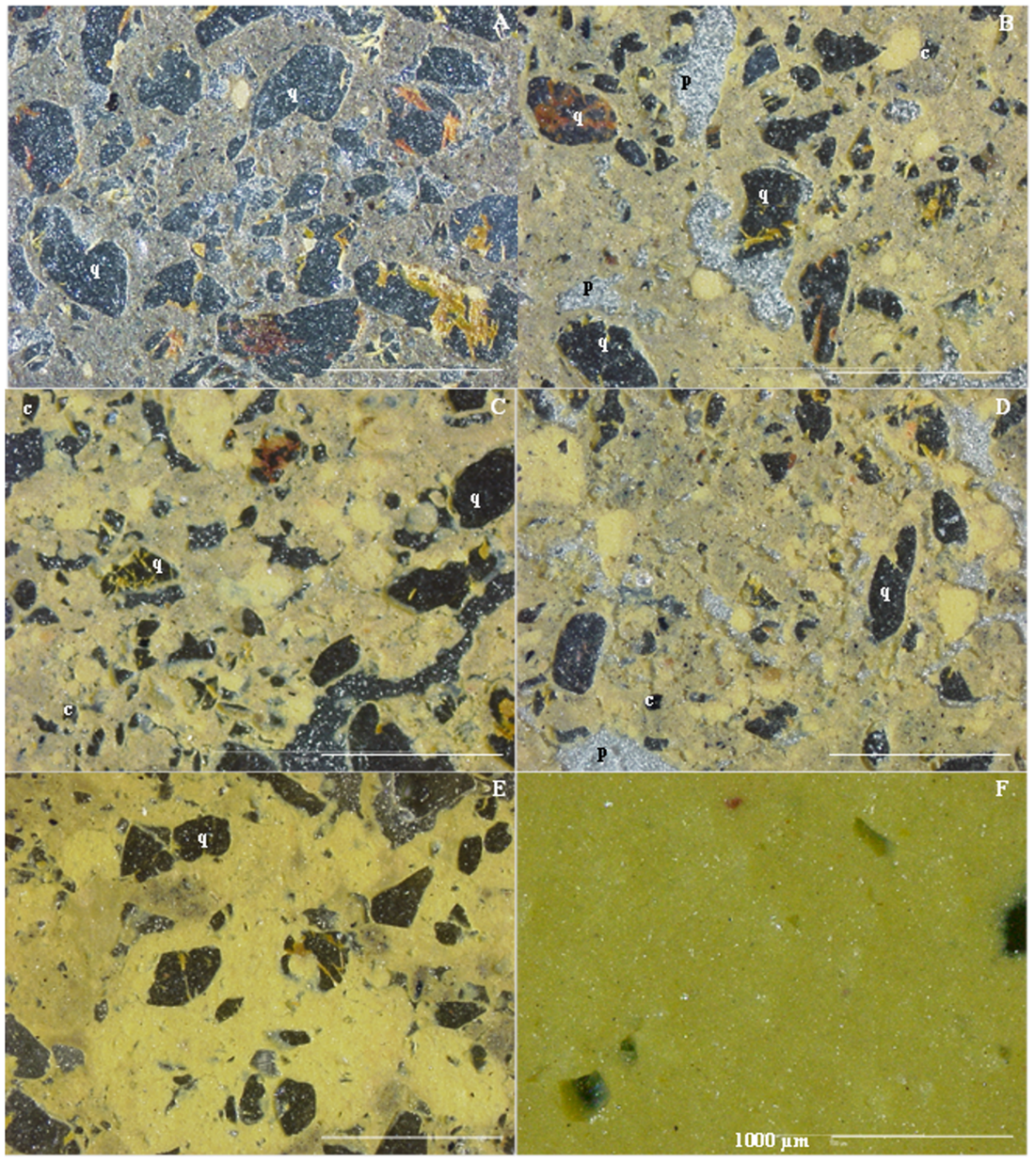
Different color of the fine material (melanization) in Pretic Anthrosol (Orthodystric, Clayic) (OIL). (A) Au_1_: single fine material dark gray (10YR 4/1); (B) Au_2_: 60% brownish yellow (10YR 6/8), 30% yellow (10YR 8/8) and 10% dark gray; (C) Au_3_: 80% brownish yellow and 20% yellow; (D) Au_4_: 45% brownish yellow, 35% dark gray and 20% yellow; (E) Au_5_: 50% yellow, 35% dark gray and 15% brownish yellow; (F) Bt_1_ exclusively yellow. q: quartz; v: voids; c: charcoal.

Very small charcoal particles (36.3 μm) were dominant in the pretic horizons. There is a direct relationship between the amount of charcoal and the groundmass color of pretic horizons. These horizons exhibit colors ranging from dark gray (10YR 4/1) to brownish yellow (10YR 6/8) in oblique incident light (OIL), with charcoal quantities of 10% and 2–5%, respectively, while areas of yellowish color (10YR 8/8) have ≤ 2% of these fragments (Fig 3).

**Fig 4:**
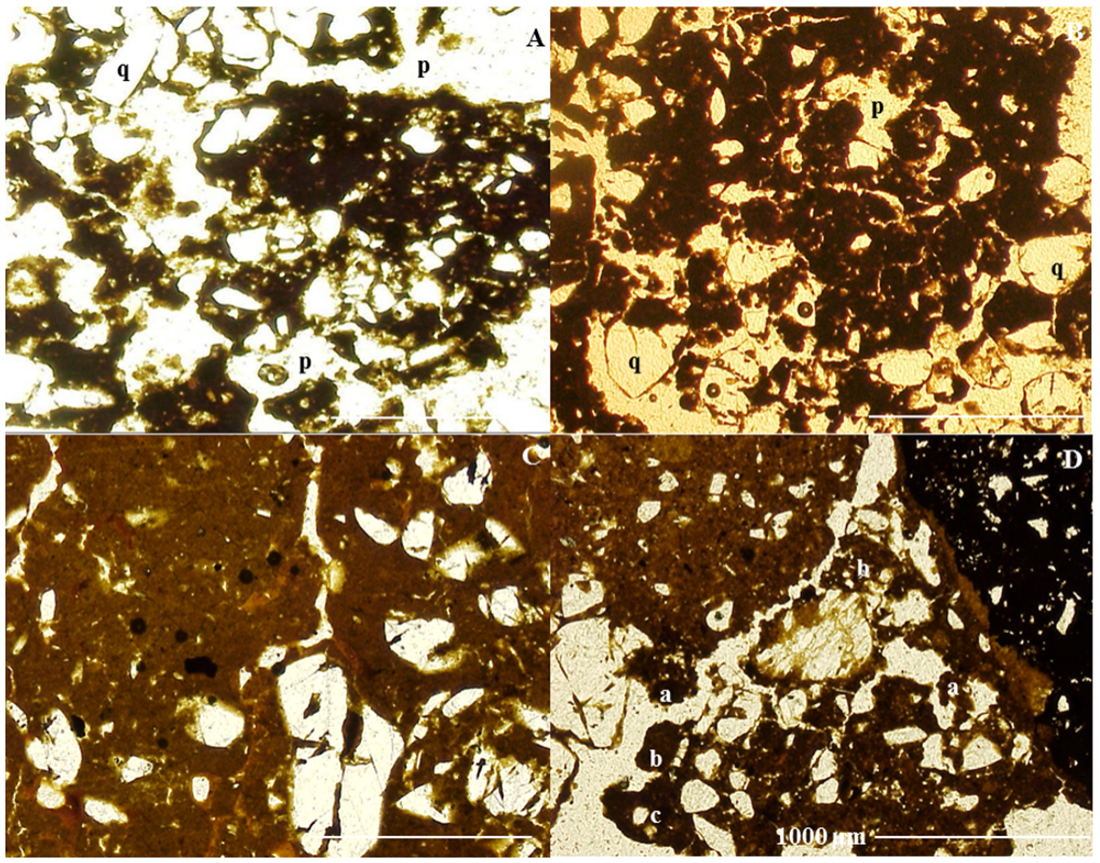
Microstructure types (PPL). (A and B) granular microstructure of Au_1_ horizons; (C) subangular block microstructure of non-anthropic A; (D) different types of microaggregates in pretic horizons. q: quartz; v: voids; a: oval microaggregates with well-sorted quartz; b: oval microaggregates with or without poorly-sorted quartz; c: polyhedral microaggregates.

The microstructure of pretic horizons is predominantly granular microaggregated (Fig 4A and 4B), while subangular blocky structure predominates in the non-anthropic A horizon. The degree of coalescence of the non-anthropic A horizon is greater than in pretic horizons, resulting in less porosity (Fig 4C). The granular peds in the pretic horizons showed small well-sorted grains of quartz (< 100 μm), different to those of larger size in the groundmass. Oval microaggregates also occur, with or without poorly sorted quartz grains and polyhedral microaggregates (Fig 4D). The structure of the subsurface horizons is massive. The number of biological channels is higher in ADE, as is the amount of packing voids in pretic horizons, while in the non-anthropic A horizon vughs predominate. Thus, the transition from an open porosity of microaggregates and blocky microstructure to a massive microstructure clearly marks the boundary between pretic and Bt horizons.

**Fig 5:**
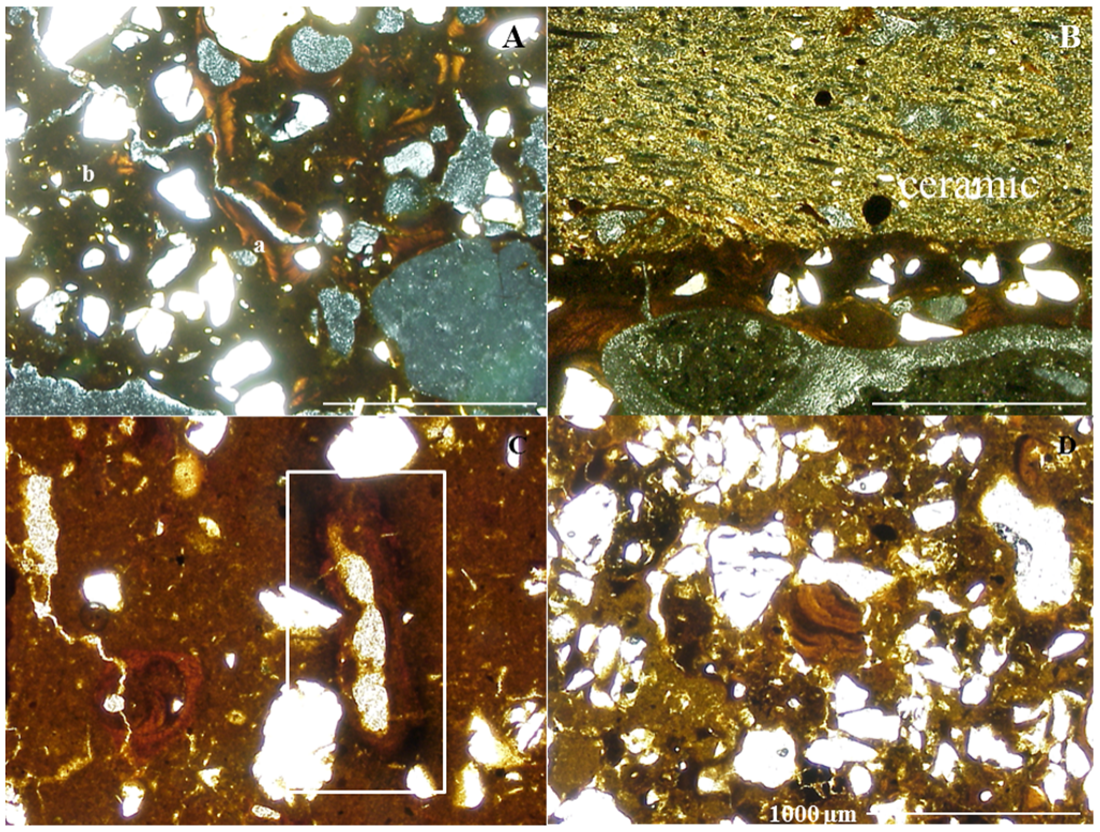
Textural pedofeatures in pretic horizons (A and B—XPL) (C and D—PPL). (A) typical yellow red microlaminated (m) and non-laminated (nl) clay coatings with strong continuous orientation and extinction bands (Au_4_); (B) illuvial clay coating on ceramic fragment; (C) non-laminated reddish yellow hypo-coating (detail); (D) fragment of clay coatings (papule) with moderately continuous orientation and diffuse extinction (Au_5_).

Pedofeatures identified in P1 and P2 are textural (coating and infilling), amorphous (nodules) and excrement. In P3 the coatings are absent and the nodules occur in trace amounts. Clay coatings in pretic are typic, microlaminated or non-laminated and, yellowish red (transmitted light) with strong continuous orientation and extinction bands (Fig 5A). These occur mainly in vughs, biological channels and fissures, but were also found on ceramic fragments (Fig 5B). The coatings are limpid (absence of charcoal or coarse material) with well-defined limits in relation to fine material, confirming the argilluviation process. The process of iron diffusion was also identified at the edge of voids through the presence of non-laminated reddish yellow hypo-coatings (Fig 5C). Fragments of clay coatings were identified in loose continuous infillings and excrement in pretic horizons (Fig 5D).

In pretic horizons and in non-anthropic A horizon loose continuous and dense incomplete infillings predominate. In pretic horizons infilling of biological channels occurs where P_2_O_5_ contents are not associated with CaO (Table 6). They consist primarily of SiO_2_ and Al_2_O_3_, with P_2_O_5_, CaO and K_2_O also occurring. In contrast, biological channels in the non-anthropic A horizon do not show significant P_2_O_5_ content and exhibit lower levels of CaO and K_2_O, as well as higher values of Al_2_O_3_, Fe_2_O_3_ and ZrO (Table 6).

**Table 6:** Chemical composition by EDS-SEM of nodules and micromass associated to biological channels in (A) Pretic Anthrosol (Orthodystric, Clayic), (B) Pretic Anthrosol (Lixic, Orthoeutric, Clayic) and (C) Haplic Xanthic Acrisol (Hyperdystric, Clayic). Central Amazon—Brazil.

Oxides	Iron nodule						Biological channels	
	Inner		Edge		Fine material			
	A^a^	Au_2_^b^	A	Au_2_	A	Au_2_	A	Au_1_^c^
	%							
**Na**_**2**_**O**	1.33	0.52	1.12	0.00	0.57	0.19	0.17	0.70
**MgO**	0.26	1.00	0.10	0.28	0.22	0.42	0.29	0.50
**Al**_**2**_**O**_**3**_	21.93	22.12	21.67	30.35	29.06	28.12	26.64	29.38
**SiO**_**2**_	29.54	31.21	27.05	56.15	55.80	54.86	55.41	57.14
**P**_**2**_**O**_**5**_	0.00	0.00	0.00	0.00	0.00	0.23	0.00	2.26
**ZrO**	5.94	6.46	6.00	4.65	5.85	6.25	9.77	n.d.^d^
**K**_**2**_**O**	1.20	1.12	1.04	0.27	0.59	0.59	0.19	0.89
**CaO**	0.38	0.41	0.38	0.29	0.34	1.31	0.33	0.50
**TiO**_**2**_	0.63	1.18	0.65	1.68	2.31	2.11	2.14	2.15
**V**_**2**_**O**_**5**_	0.07	0.05	0.19	0.05	0.00	0.00	0.06	n.d.
**MnO**	0.00	0.09	0.10	0.00	0.03	0.05	0.04	n.d.
**Fe**_**2**_**O**_**3**_	38.68	35.66	41.59	6.22	5.16	5.84	4.90	6.47
**CuO**	0.05	0.17	0.11	0.06	0.07	0.03	0.08	n.d.

^a^ non-anthropic A horizon (P3)

^b^Au_2_ (P2)

^c^Au_1_ (P1)

^d^ n.d.: not detected

**Fig 6:**
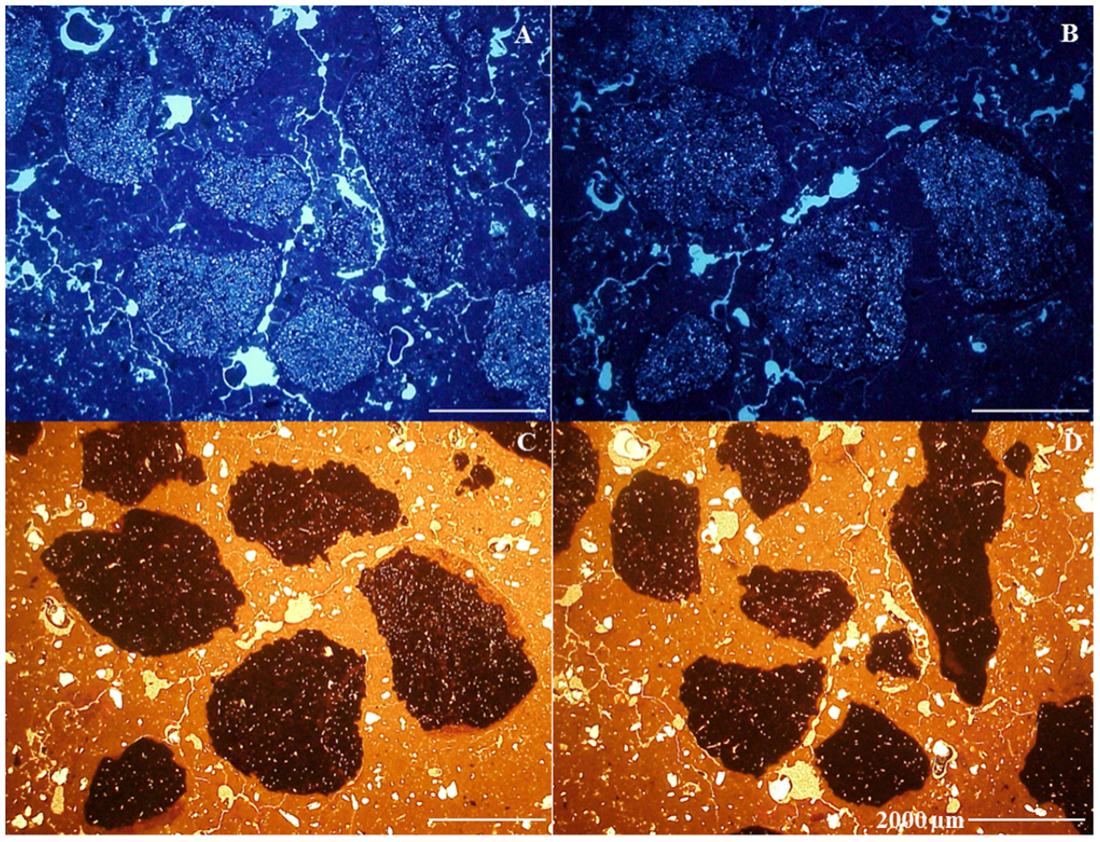
Amorphous pedofeatures (nodules) in Pretic Anthrosol (Lixic, Orthoeutric, Clayic). (A and B) irregular, orthic and concentric nodules with very small voids, spongy-like (ultraviolet light); (C and D) nodules formed of several smaller entities each separated by adjacent groundmass (disjointed morphology) (OIL).

Nodules were present in all the horizons from P2. In P1, the nodules occur from Au_1_ to Au_4_, while in P3, only a single nodule was identified in the A horizon. In transmitted light the nodules are black (10YR 2/1) while under OIL they are red (2.5YR 4/6) to yellowish red (5YR 5/8), suggesting the presence of hematite and goethite. They are irregular, orthic, typical and concentric with very small voids, resulting in a sponge-like appearance (Fig 6A and 6B). Nodules with or without fine sand-sized quartz grains and nodules formed of several smaller entities, each separated by adjacent groundmass, predominate (Fig 6C and 6D).

**Fig 7:**
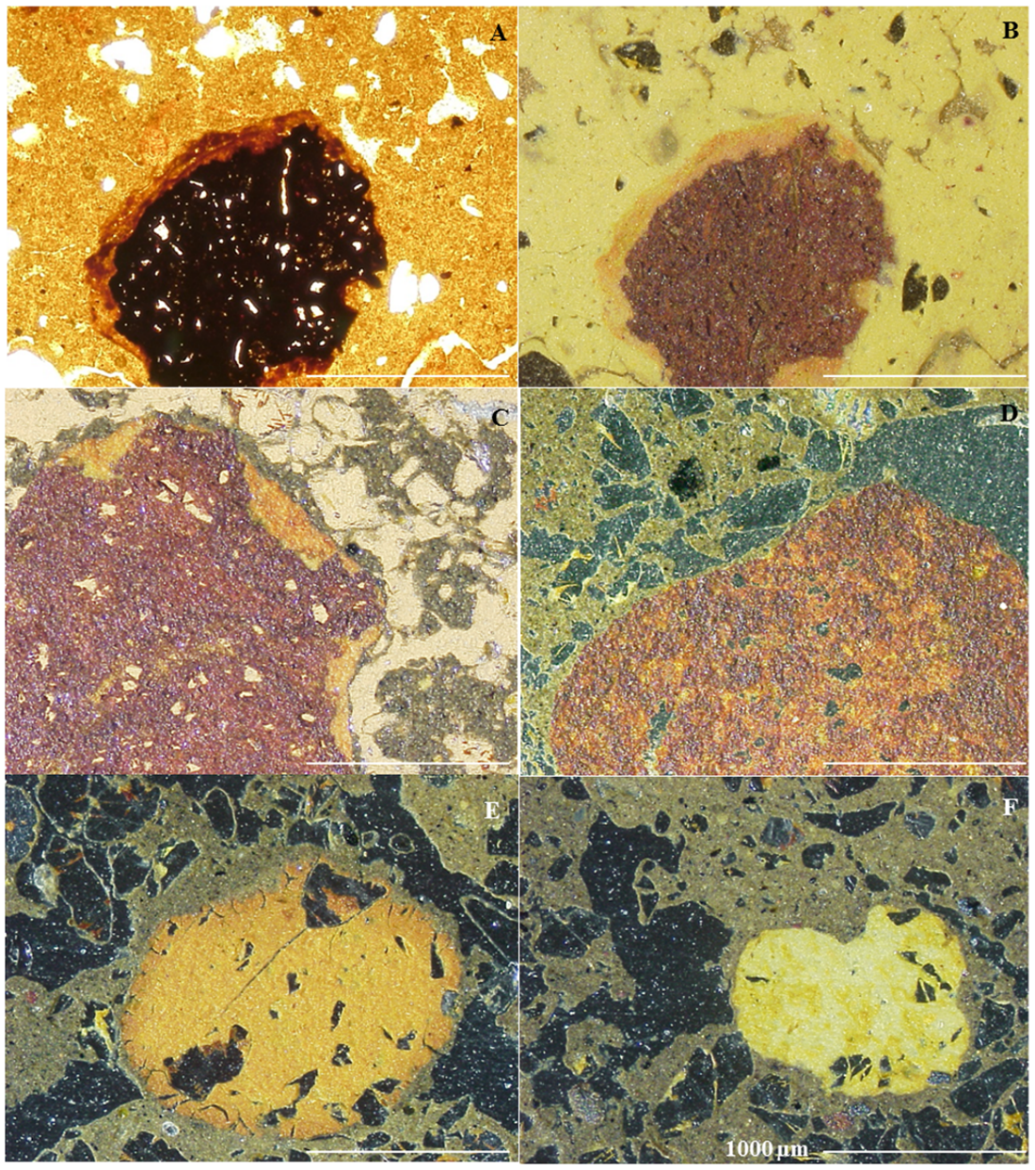
The xanthization process at different stages in the nodules. (A and B) dark red (PPL) and yellow (OIL) nodules with a strongly impregnative core and a weaker impregnative cortex (dissolution); (C) detail of nodule with internal fabric severed at the boundaries with groundmass and serrated edges (degradation) (OIL); (D) hematite nodule at intermediate degradation stage with yellowish red infillings; (E) highly changed nodule, predominantly yellow with yellowish red edges (OIL); (F) nodule completely changed, yellowish and without reddish material (OIL).

With the exception of the non-anthropic A horizon, all nodules present an internal fabric severed at the boundaries with groundmass, serrated ends (Fig 7A and 7B), polyconcave vughs and cracks, yellowish red infillings and some quartz fractured and impregnated with yellowish material. In pretic horizons, the concentration of SiO_2_ and Al_2_O_3_ increases in the nodule-soil matrix direction, whereas contents of Fe_2_O_3_ decrease. Conversely, the nodule in the non-anthropic A horizon does not show depletion of Fe_2_O_3_ (Table 6). Deferrification of nodules in pretic horizons (transformation from hematite to goethite) was observed at different stages in the nodules (Fig 7C–7F): i) hematitic nodules at an intermediate stage of degradation with yellowish red infillings; ii) significantly changed nodules, predominantly yellow with small yellowish red parts; iii) completely changed nodules, yellowish without reddish material.

## Discussion

### Soil morphology and physico-chemical attributes

Phytolith studies show that the greater thickness of ADE in P1 indicates more intensive use of the soil, probably related to the domestic context [21]. The darker color of horizons Au_1_ and Au_2_ can be credited mainly to the incorporation of organic remains (e.g. palm leaves, bones and charcoal) [21], [23], while the brown color of horizons Au3—Au_5_ results from mixing with aggregates from Bt horizons, conferring a coloration similar to that of the original soil matrix. Red mottles and plinthite occur due to saturation events with oxygen-depleted (anaerobic) water, indicating reduction, translocation and oxidation of iron. In the petroplinthic horizon (Btcf), past conditions of lower humidity promoted the hardening of plinthites.

The granular structure and weakly developed blocky structure of pretic horizons and non-anthropic A horizon results from higher OM content and the greater intensity of bioturbation, while the large blocks with a moderate to strong degree of aggregation of subsurface horizons is in agreement with lower root and fauna activity and a kaolinitic mineralogy with low oxide content. The clay skins observed in the field in some pretic horizons corresponds to argilluviation coatings (confirmed by the micromorphology), while those present in subsurface horizons from P3 reflect particle rearrangement on the surfaces of the structural units.

Anthropic activities changed the texture of AB horizons (P3) from clayey to sandy clay loam (Au_1_—P1). This change is probably due to the fusion of clay and organic matter into sand-sized particles by fire [32]. However, our study shows that strong argilluviation may have played a key role in this phenomenon. Chemical changes in non-anthropic subsurface horizons from ADE result from biological activity that incorporated chemically richer material from pretic horizons at depth.

### Melanization and cumulization

The widespread charcoal fragments in the pretic horizons influence melanization and cumulization. The greater quantity and smaller size of charcoal in pretic horizons compared to non-anthropic horizons (Fig 2) is a result of prolonged and continuous use of the soil by humans (anthrosolization). The dark color results from the concentration of charcoal particles and resilient substances derived from pyroligneous residue from the carbonization process [33]. Studies on ADE show that microscopic charcoal fragments indicate efficient biological comminution, besides reflecting soot particle concentration on the interior of residential structures [10].

Melanization of the anthropic horizons is intimately related to the proportion of charcoal micro-particles. Through anthropic incorporation of these fragments and subsequent homogenization through biological activity, horizons with fine material that are yellow, brownish yellow and yellowish red (e.g. AB horizons and Bt horizon) become yellowish brown and dark gray (pretic horizons). Due to pretic horizons presenting different quantities of charcoal and, as a consequence, fine material of different colors, they also exhibit macromorphologically different levels of melanization. Thus, pretic horizons that present exclusively dark gray fine material present as black under field conditions, while horizons of more than one color observed under the microscope are yellowish brown to dark brown.

In contrast to other studies on ADE [10], [11], the melanization of the studied pretic horizons is not related to the occurrence of manganese nodules. These nodules were not found on thin sections, nor were they identified through effervescence tests in the laboratory. Our results also show that melanization of the pretic horizon is not directly related to the quantity of organic matter, considering that the non-anthropic A horizon, which possesses higher content of organic matter when compared to the pretic horizon, does not present considerable melanization. Therefore, in relation to melanization and cumulization processes, the main micromorphological difference between pretic horizons and non-anthropic horizons is the higher quantity and smaller fragment size of charcoal in ADE

The stability of organic matter in ADE has often been put down to the high content and recalcitrance of black carbon [34]. The molecular characterization of different fractions of OM from the studied profiles demonstrates that pretic horizons have a higher contribution of BC in the humin fraction [35]. These differences suggest greater BC stability in ADE, which is probably related to differences in quantity and quality of BC and/or the association of BC with inorganic compounds [35], [36]. Thus, as demonstrated by the micromorphological evidence, charcoal is an important component of pretic horizons, because by representing the intact fraction of BC and by constituting finely divided particles, it produces a considerable effect on pigmentation (melanization) and contributes to the increased stability and accumulation of OM in ADE (cumulization).

### Biological action

An important effect of bioturbation is the fragmentation of charcoal and the distribution of the fragments to the subsurface horizons, contributing to the melanization and thickening of pretic horizons. The smaller amount and greater size of charcoal fragments in Bt horizons, indicating favorable conditions for its preservation, strengthens this hypothesis. The bioturbation is also crucial to the melanization of transitional and subsurface horizons. In a thick A horizon of Ferralsols, aggregates larger than 1 mm were impregnated by microfragments of charcoal scattered over the groundmass by the fauna and probably also by degradation, increasing the melanization effect of the soil [33]. Bioturbation in pretic horizons is also responsible for the increased number of active channels facilitating the diffusion of air, water percolation and translocation of materials.

One of the origins of the granular microstructure of ADE is zoogenetic [37]. Micromorphological evidence showed that granular peds are similar in size, shape and internal structure to excrement produced by termites and/or ants present within biological vughs and channels. These granules also show small well-sorted grains of quartz (< 100 μm), different from those of larger size in the groundmass (Fig 4D). In addition, the presence of oval microaggregates, with or without poorly sorted quartz grains and the presence of polyhedral microaggregates (Fig 4D), respectively indicates either biological or geochemical genesis, or strictly geochemical genesis [38]. This microstructure, consisting of a mixture of organo-mineral aggregates with similar structure to the chernic horizon, was identified in other ADE [3], [10], [14]. The subsurface horizons present massive microstructure due to coalescence of microaggregates [38] and the face-to-face arrangement of kaolinitic fine material, with low content of Fe oxides [39]. In addition, the transformation of biogenic apatite through ingestion by earthworms and small arthropods results in the accumulation of P-organic and P-Al (P contents not associated with Ca), which are residual in the soil [40]. As such, together with biological channels in Bt horizon being filled with material from the pretic horizon, this shows an intense mixture of material (thickening of pretic horizons) and favors chemical enrichment of subsurface horizons.

### Argilluviation

Dusty illuvial clay coatings with low birefringence were observed in planar pores and vughs in anthropic horizons of ADE, suggesting ash deposition [10]. Illuvial clay coatings were also identified in the studied anthropic horizons and were found in quantities > 2% (Fig 5A). According to WRB [6], 1% of these features characterize an argic horizon. The non-classification of these horizons as exclusively B horizon is attributed to the presence of archaeological material, dark color, calcium and phosphorus enrichment and the occurrence of unusual minerals in soils of the region. Therefore, it is suggested that these horizons be identified as BAtu, rather than simply Au or Bt horizons.

The observation of truncated clay coatings in horizon B, immediately below horizon A of an Anthrossol prepared for agricultural activity (Terra Mulata), suggests that the “Terra Mulata” has not been formed *in situ*, but has been intentionally deposited [11]. According to the author, this configuration results from the intervention of digging tools or from the removal of the organic surface horizon, suggesting scraping, raking, or churning of the soil. Similar features were found in the studied pretic horizons, here characterized as clay coatings with moderately continuous orientation (Fig 5D). Due to having occurred in dense continuous infillings and/or being associated with excrement, as well as features denoting soil movement or burial of horizons through human activity not having been found [10], [11], these features were interpreted as argilluviation coatings degraded by bioturbation.

The occurrence of clay coatings with moderately continuous orientation in active pores and on ceramics demonstrates that the process of argilluviation is current (Fig 5B). The higher amounts of water-dispersible clay in pretic horizons contribute to this process. The presence of carboxylic radicals and the higher O/H ratio of humic acids in pretic horizons increase the electronegativity of ADE [41]. Under these conditions the aggregates become less stable and hinder colloid flocculation. In contrast, the absence of clay coatings in P3 indicates genesis unrelated to the argilluviation process. Due to the kaolinitic mineralogy, this soil has low density of negative charges. Under these conditions, Al^3+^ predominates in the exchange complex and promotes flocculation. Thus, the superficial removal of the fine fraction from the topsoil through erosion (elutriation) is the process responsible for the textural differentiation in this soil.

### Iron nodule dissolution

Lateritic mantle covers in dismantlement, give rise to degradation pisolites, characterized by the occurrence of goethitic cortex [16], demonstrating that the current conditions diverge from those in which the Fe nodules were formed [42]. Despite the occurrence of this cortex in nodules from subsurface petroplinthic horizons (P2), the micromorphological evidence demonstrates that the thickness of this cortex is significantly larger in nodules from pretic horizons, indicating intensification of hematite alteration in goethite in these horizons.

The iron oxide hypo-coatings in aggregate walls and active channels in the pretic horizons confirm the establishment of local redoximorphic conditions [43]. The lack of identification of iron reduction characteristics in the groundmass (e.g. iron depletion hypo-coating) indicates that the saturation is temporary [44], related to the process of episaturation, which is common in soils with a very clayey texture, with a blocky structure and subject to elevated levels of precipitation (> 2000 mm/year). During periods of intense and frequent rainfall, the lower part of the pretic horizon remains saturated for longer and, due to the incorporation of more humid OM at depth in ADE, the microbial activity promotes the reduction of Eh values, which compete for the reduction of iron oxides at the edges of petroplinthite nodules. An increase of reductive dissolution of Fe-oxides with the addition of OM was also reported from other archaeological sites [45], [46]. The deferrification of iron nodules in pretic horizons is important for the following: i) hematitic core reduction at the expense of the growing cortex, which explains the presence of concentric nodules; ii) release of Al and Si into the soil matrix, increasing stability of fine kaolinitic material; iii) clay source and; iv) contribution to the xanthization process, increased in humid conditions and in the presence of organic C.

Nodules with irregular external morphology are indicative of origin *in situ* [47]. The predominance of nodules with or without quartz grains indicates formation by Fe oxidation and nucleation in a clay matrix similar to the adjacent fine material. Yellowish red infillings in biological channels inside the nodules indicates the initial action of bioturbation and subsequent ferrugination of the porosity. In addition, the occurrence of nodules formed by several smaller entities, each separated by adjacent groundmass, indicates formation from the degradation of other larger nodules (Fig 6C and 6D). All this micromorphological evidence points to autochthonous nodule formation. Therefore, we postulate that reduced iron arising from more elevated parts of the landscape precipitated as oxide in the lowest parts. Initially mottles were formed, which evolved into plinthites and petroplinthites through lowering of the water table and are currently found in dismantlement, shown by individualization in pisolites with goethitic cortex. These assertions suggest that the petroplinthite nodules originated during paleoclimatic or geomorphological conditions distinct from the current conditions.

## Conclusions

This study confirms that the anthropogenic sedimentation model allied to the process of soil homogenization through bioturbation explains ADE formation at the Caldeirão site. Moreover, it corroborates previous studies that credited melanization and SOM stabilization (cumulization) of ADE to the occurrence of charcoal. We also confirmed that the translocation of charcoal to subsurface horizons through bioturbation transformed subsurface horizons into anthropic horizons, besides contributing to subsurface chemical enrichment of the ADE.

Amplifying the knowledge on previously described processes in ADE, we showed that pretic horizons presented distinct melanization levels that are directly related to the quantity of charcoal, and therefore not related to their mineralogical composition, as reported in previous studies. Furthermore, besides the highlighted contribution to homogenization of horizons and formation of soil aggregates, bioturbatiom may also fragment pedological features indicative of argilluviation (e.g papules), possibly leading to different interpretations in respect to their genesis.

Additionally, and surprisingly, this study also showed that anthropic activities intensified the degradation of iron nodules in ADE, increasing the clay content in the soil and contributing to the xanthization process. Subsequently, nodule degradation contributed to destabilization of the structure, and combined with the anthropic increase of soil electronegativity, promoted argilluviation, a process currently observed in coatings of ceramic fragments.

Therefore, the novelty from this study was to broaden current knowledge in respect to the genesis of ADE, confirming the hypothesis that pedogenetic processes previously credited to natural causes may have been installed and intensified through ancient anthropic activities. Moreover, besides contributing to updating the concept of pretic horizon on the WRB, this study increases current archaeological debate by showing that if the formation of the ADE involved the addition of soil material, then this corresponds to the surrounding soil, that is, it did not involve the addition of soils from diverse origins (as postulated from the floodplains with different sedimentation). The study also shows that a stratigraphic pattern in the disposal of waste cannot be identified, a hypothesis which cannot be discarded given the homogenization of the soil horizons through.
